# Prevalence of Gastric Cancers among Patients Undergoing Upper Gastrointestinal Endoscopies in a Tertiary Care Hospital in Nepal: A Descriptive Cross-sectional Study

**DOI:** 10.31729/jnma.5657

**Published:** 2021-01-31

**Authors:** Subash Bhattarai, Merina Gyawali, Sudeep Regmi

**Affiliations:** 1 Department of Medicine, Manipal College of Medical Sciences and Teaching Hospital, Pokhara, Nepal; 2 Department of Radiodiagnosis and Imaging, Manipal College of Medical Sciences and Teaching Hospital, Pokhara, Nepal; 3 Department of Pathology, Manipal College of Medical Sciences and Teaching Hospital, Pokhara, Nepal

**Keywords:** *adenocarcinoma*, *endoscopy*, *gastric cancer*, *histopathology*

## Abstract

**Introduction:**

Gastric cancer is a common malignancy of the upper gastrointestinal (UGI) tract. Gastric cancer is a common cause of death worldwide. This research aimed to study the prevalence of gastric cancer among patients undergoing upper gastrointestinal endoscopies.

**Methods:**

A descriptive, cross-sectional study was conducted in the Department of Medicine at Manipal Teaching Hospital, Nepal, from January 2018 to June 2020. A total of 2640 subjects underwent upper gastrointestinal endoscopies over the study period. Ethical approval was taken from the institutional review committee of Manipal College of Medical Sciences (MEMG/ IRC/ 383/ GA). Data were analyzed by SPSS version 20.

**Results:**

The prevalence of gastric cancer among patients undergoing UGI endoscopies was 2.4%. The mean age of subjects was 58 ± 12.35 years (range of 31 to 96 years) with male predominance (M: F=1.9:1). Antrum was the most common site for gastric carcinoma. The most common morphology was ulcerative growth (46.9%). Adenocarcinoma (98.4%) was the most common histology, and the majority was of intestinal subtype (56.3%).

**Conclusions:**

Gastric cancer is not an uncommon finding in patients undergoing UGI endoscopies. Gastric cancers were commonly seen above 50 years of age and predominant in males. Patients with gastric carcinoma usually present late with advanced disease stages and unfavorable histopathology

## INTRODUCTION

Cancer is one of the most dreaded non-communicable diseases that have become an important contributor to disease's global burden.^1^ The gastrointestinal (GI) cancers are responsible for more cancers and more deaths from cancer than any other cancers.^2^ Gastric cancers is the commonest upper gastrointestinal (UGI) malignancy and is the second most common cause of death worldwide.^3^

A high incidence of gastric cancer has been reported from Southeast Asia attributed to the high consumption of preserved food containing carcinogenic nitrates.^4^ Endoscopy followed by endoscopic biopsy is a very useful diagnostic tool for pathologies of the upper GI tract, including gastric cancers.^5^

National data on the demographic profile of stomach cancers are scanty. This research is meant to study gastric cancer prevalence among patients undergoing UGI endoscopies attending medical gastroenterology clinic at a tertiary care Teaching Hospital in Gandaki province, Nepal.

## METHODS

This was a hospital-based, observational, crosssectional, retrospective study carried out in the unit of Medical Gastroenterology, Department of medicine at Manipal College of Medical Sciences and Teaching Hospital, Nepal, from January 2018 to June 2020 for a period of 30 months. The Institutional Review committee of the Manipal College of Medical Sciences approved the study (MEMG/IRC/383/GA/26th Aug 2020). The sample size was collected using the formula,

$$\begin{array}{ll} \text{n} & ={\text{Z}}^{\text{2}}\text{pq}/{\text{e}}^{\text{2}} \end{array}$$

Where,

Z = 1.96 (at 95% confidence interval)
p = taking 50%
e = margin of error, 5%

The calculated minimum sample size was 385.

Cases from January 2018 to June 2020 were studied from the records of the Endoscopy unit, departmental admission, and discharge summaries. All consecutive cases of 18 years and above who presented to the Medical Gastroenterology unit (under the department of medicine) either as outpatients or in patients who underwent upper GI endoscopy were enrolled for the study. Patients already diagnosed with gastric carcinoma or those with no histopathological confirmation or those with incomplete records, those with primary gastric lymphoma, gastrointestinal stromal tumor, and neuroendocrine tumor were excluded from the study. Studied variables include endoscopic findings, sites of lesions, and histopathological subtypes.

Contrast-Enhanced Computed Tomography (PHILIPS Ingenuity 128 CT Scanner, NETHERLANDS Inc) scan of the abdomen and pelvis was done to complete the evaluation of upper GI tract and study of regional lymphadenopathies, ascites, and metastases. After hemodynamic stabilization, overnight fasting and pre-medications, and informed consent, an endoscopy (PENTAX EPK 700, PENTAX JAPAN Inc) was performed. Endoscopy findings were noted. At least 4 tissue biopsies were taken from the nonnecrotic gastric pathological site in 10% formalin and sent to the pathology department for histological assessment. The histologic tumor type of gastric cancer (GC) was classified according to the Lauren classification. (Intestinal, diffuse, and mixed type). The microscopes used were Carl Ziess Axiostar Plus and Olympus CX21. Endoscopy findings with biopsies from the suspected sites followed by histopathology were all helpful in the diagnosis of gastric cancer.

Data collected were collected in a preformed sheet. Data were entered in Microsoft Excel 2010 and analysis was done using SPSS version 20. All categorical data were expressed in percentage and absolute number. All continuous numerical data were expressed in mean ±SD. All tests were analyzed with a 95% confidence interval.

## RESULTS

A total of 2640 subjects underwent upper GI endoscopies over the study period of 30 months. Sixty-four patients were detected with gastric cancer, demonstrating a prevalence of 2.4% among patients undergoing UGI endoscopies. The study group comprised of 42 (65.6%) males and 22 (34.4%) females (M: F=1.9:1). The mean age of subjects was 58±12.35 years with a range of 31 - 96 years of age. Four (6.2%) patients were aged below 40 years of age. Patients were classified as per age group (Table 1). A majority (n=36; 56.25%) of cases were aged between 51 and 70.

**Table 1:** Age groups distribution of gastric carcinoma (n = 64).

Age Groups	Numbers	Frequency (%)
≤50 years	12	18.75
51-70 years	36	56.25
>71 years	16	25
Total	64	100

UGI Endoscopy revealed that antrum (48.4%) was the commonest site of gastric carcinoma. The other locations were as in Table 2. Gastric carcinoma were observed commonly with ulcerative lesions (n=30; 46.9%), followed by ulceropoliferative (n=24, 37.5%) and proliferative growth (n=10; 15.6%).

**Table 2:** Distribution of Gastric carcinoma (n=64).

Site	Numbers	Frequency (%)
Antrum	31	48.4
Antrum + Body	15	23.4
Body	10	15.6
Body + Fundus	5	7.8
Fundus	3	4.7
TOTAL	64	100

CECT of the abdomen revealed findings suggestive or suspicious of gastric malignancies in 46 (71.8%) patients. CT revealed mild to moderate ascites in 12 (18.7%) cases. The liver was the most common site of metastasis found in 16 (25%) patients, followed by left supraclavicular lymph node (12.6%), peritoneal metastasis (10%), and multiple metastases (8.1%). Majority, 30 (46.8%) of these patients presented with Stage III carcinoma followed by stage IV (n=16; 25 %), stage II (n=12; 18.8%) and Stage I (n=6; 9.4%).

After the endoscopic biopsy, the most common histology was adenocarcinoma seen in 63 (98.4%), followed by a single (1.6%) adenosquamous carcinoma case. Among the adenocarcinomas, 36 (56.3%) were intestinal type, 16 (25%) were diffuse, and the rest, 12 (18.7%), were mixed adenocarcinoma. Most (n=22; 61.1%) of the intestinal carcinoma were well to moderately differentiated whereas, most of the diffuse or mixed type tumors were poorly differentiated (n=20; 71.4%).

## DISCUSSION

In the current study, the prevalence of gastric cancer among patients undergoing UGI endoscopies was 2.4%. In a previous Nepalese study from this region in 2013 comprising of 2820 endoscopies by Shrestha et al.^6^, gastric carcinoma prevalence was reported to be 1.8%. The prevalence or detection of gastric cancer may be rising every year in this region.

The mean age of subjects was 58±12.35 years with male predominance (M: F=1.9:1). Majority (56.3%) of cases were aged between 51-70 years of age. In a previous Nepalese study by Ghosh et al.^7^, gastric tumors were seen even more (83%) in the same age group of 51-70. The mean ages were 57, 54, 54, and 51 years by Das et al.^8^, Cherian et al.^9^, Debbarma et al.^10^, and Kabir et al.^11^, respectively. These studies have mentioned that gastric carcinoma is commonly observed between 50 and 60 years of age with male predominance. In the current study, 6.2% of patients were aged below 40 years of age. In a study in Vietnam by Quach et al.^12^, gastric cancer incidence in young below 40 years of age was even higher (16.3%). So, this cancer is not only restricted to the elderly.

Antrum (48.4%) was the commonest site of gastric carcinoma, followed by carcinoma of the body in the current study. Antrum was similarly found to be the most common site of carcinoma according to Ghosh et al.^7^ (70% ), Mir et al.^13^ (57.52%), Barad et al.^14^ (50.6%), Saha et al.^15^ (51.9%), and Kabir et al.^11^ (50%). In the current study, gastric carcinoma was observed commonly with ulcerative lesions (46.9%), followed by ulceropoliferative (37.5%) and proliferative growth (15.6%). Saha et al.^15^ had similar findings with ulcerative lesions most commonly (57.8%) followed by ulceroproliferative (24.9%) and polypoidal lesion (17.3%). In the study by Kabir et al.^11^, ulcerative (56%) was similarly the commonest growth followed by polypoidal (34%) and ulceroinfiltrative (10%) lesions.

Majority of these patients presented with Stage III carcinoma (46.8%), followed by stage IV (25 %), stage II (18.8%), and Stage I (9.4%) in the present study. Most of the patients in the study by Barad et al.^14^ in India also presented in stage III (62.7%). More than two-thirds of the patients were diagnosed with advanced gastric cancer in a study in Iran.^16^ Patients presented with advanced Stages III followed by stage IV even in the studies by Isobe et al.^17^ in Japan and Park et al.^18^ in South Korea. All these studies suggest that patients with gastric carcinomas usually present late and with advanced stages in Asian countries.

The most common histological morphology was intestinal adenocarcinoma (56.3%) in our study, consistent with other studies.^14,18,19^ Intestinal type adenocarcinoma was similarly the commonest histology and was seen in 55.9% by Eskandar et al.^16^, 53.6% by Saha et al.^15^, 53% by Ghosh et al.^7^, and 52% by Kabir et al.^11^ Most (61.1%) of the intestinal carcinoma were well to moderately differentiated whereas, most of the diffuse or mixed type tumors were poorly differentiated (71.4%). Similar were the findings by Barad et al.^14^ All these studies suggest that patients with gastric adenocarcinomas usually present with advanced morphology and unfavorable histopathology.

This study had its limitations. It was a retrospective study with no follow-ups. Clinical presentation, operability, and clinical outcomes of gastric cancers were not studied. The study reflects the findings of a specific geographical area.

## CONCLUSIONS

Gastric cancers are not uncommon findings in patients undergoing UGI endoscopies. They were commonly observed in patients aged more than 50 years of age. Antrum was the most common site. The majority presented with ulcerative growth on endoscopy and advanced stages in CECT abdomen with unfavorable histopathology. Thus, it is recommended that when alarming features and chronic symptoms are present, a delay should not be made for evaluation with upper GI endoscopy. Biopsy of the pathological lesions followed by histopathological confirmation must be done to rule out UGI tract malignancies.
